# Use of dexmedetomidine in critical-ill patients: is it time to look to the actual evidence?

**DOI:** 10.1186/s13054-023-04618-z

**Published:** 2023-08-29

**Authors:** Orlando R. Pérez-Nieto, Rafael A. Reyes-Monge, Ignacio Rodríguez-Guevara, Eder I. Zamarrón-López, José A. Meade-Aguilar

**Affiliations:** 1 Intensive Care Unit, Hospital General de San Juan del Río, Querétaro, Mexico; 2 Department of Anesthesia, Hospital General de San Juan del Río, Querétaro, Mexico; 3 Critical Care Department, Hospital Regional General IMSS No. 6, Ciudad Madero, Tamaulipas Mexico; 4 Department of Medicine, Boston University Chobanian and Avedisian School of Medicine, 72 E Concord St, Boston, MA USA

**Keywords:** Dexmedetomidine, Sedation, Critical-ill patients, Mortality, Delirium, Warning statement, Intensive care unit

To the editor

Dexmedetomidine is an alpha-2 (ɑ2) adrenergic receptor and imidazoline type 2 receptor agonist that received Food and Drug Administration (FDA) approval in 1999 as a sedative and analgesic that also reduces stress, anxiety, and the risk of delirium in critically ill patients. Commercialized under the name *Precedex* by Pfizer, it has been used widely across the globe, and it´s still considered to be part of a gold-standard care agent alongside propofol for critically ill patients, patients in the operating room and patients under mechanical ventilation [1]. However, robust evidence published in the last years show serious concerns about its safety and efficacy as a sedative agent, leading to the debate about its recommended usage.

The SPICE III was a randomized controlled trial that included 4000 patients and evaluated the mortality at the ICU when administering dexmedetomidine at a maximum dose of 1.5 mcg/kg/h adjusted to reach a targeted Richmond Agitation and Sedation Scale (RASS) score of − 2 to + 1, compared to usual care involving propofol and benzodiazepines [2]. The study reported no significant difference in 90-day mortality (95% CI − 2.9 to 2.8, *p* = 0.98). However, trying to reach the targeted RASS score, the dexmedetomidine group needed more sedative agents when compared with the control group, reflecting a potential and considerable efficacy issue of the agent. Added to this, the dexmedetomidine group experienced a significantly higher incidence of adverse effects including bradycardia (5.1% vs. 0.5%, *p* =  < 0.0001), hypotension (2.7% vs. 0.5%, *p* =  < 0.0001), and persistent asystole (0.7% vs. 0.1%, *p* = 0.003), indicating a potential major flaw of the indiscriminate use of dexmedetomidine. A subgroup analysis revealed varied treatment responses based on age, with a significant higher mortality observed in patients younger than 63.7 years. Although exploratory results from subgroup analysis should be taken cautiously, this might represent the opening of an unprecedented and unexpected pandora box in one of the most common sedative strategies worldwide.

Consequently, a post-hoc Bayesian analysis that included 3905 ventilated critical ill patients confirmed the subgroup analysis results [3]. The analysis suggested that the utilization of dexmedetomidine may have a higher probability of 90-day mortality in patients aged ≤ 65 years (OR 1.26, 95% CI 1.02–1.56) with a 98.5% probability of harm when compared to usual care sedation.

Added to this, another sub-analysis of SPICE III explored the association between different infusion doses of dexmedetomidine and propofol, as well as their relationship with mortality, with consideration for age [4]. The findings indicated that increasing the dexmedetomidine dose was associated with elevated 90-day mortality in younger (age < 65 years) patients (HR 1.3, 95% CI 1.03–1.65, *p* = 0.029).

Finally, a systematic review and meta-analysis of controlled randomized trials evaluated the use of dexmedetomidine in patients with sepsis reported a lower incidence of mortality associated with dexmedetomidine compared to any other intervention (RR 0.83, 95% CI [0.69, 0.99]) [5]. Nonetheless, this reduction in mortality was only consistently observed when compared only to benzodiazepines (RR 0.36, 95% CI [0.18, 0.70]), but not with propofol (RR 0.89, 95% CI [0.74, 1.07]). Dexmedetomidine was also not found to be associated with a lower risk of delirium compared to other sedatives (RR 0.98, 95% CI [0.72, 1.33]). Additionally, dexmedetomidine did not demonstrate a reduction in ICU days compared to other sedatives (SMD − 0.22, 95% CI [− 0.85, 0.41]), nor did it reduce the duration of mechanical ventilation (SMD 0.12, 95% CI [− 1.10, 1.35]), or increase ventilator-free days; MD 1.68; 95% CI [− 1.50, 4.85]. In addition, dexmedetomidine presented a higher risk of arrhythmias (RR 2.69, 95% CI [1.19, 6.08]), but not hypotension (RR 1.04, 95% CI [0.46, 2.36]).

Although dexmedetomidine showed promise as a sedative, there are current concerns that should not be overlooked regarding its use. Figure 1 illustrates the timeline of relevant events related to the use of dexmedetomidine in critically ill patients. The results of SPICE III even led to the issuance of a health alert in Europe (https://www.ema.europa.eu/en/medicines/dhpc/dexmedetomidine-increased-risk-mortality-intensive-care-unit-icu-patients-65-years.) and New Zealand (https://www.medsafe.govt.nz/safety/DHCPLetters/DexmedetomidineFebrruary2023.pdf.) about the risks of using dexmedetomidine. The commercialization of dexmedetomidine represents a millionaire market for the pharmaceutical industry and this should not overshadow the current evidence regarding its efficacy and safety. Still, further studies are needed to determine if dexmedetomidine can still be recommended in particular scenarios and in definitive, task force groups are needed to take action based on the currently published data.

**Fig. 1:**
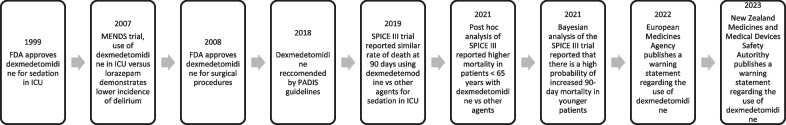
Timeline of key milestones in the use of dexmedetomidine in critically Ill patients

## Data Availability

Not applicable.
